# Intermittent Fever

**Published:** 1882-02

**Authors:** C. F. Millspaugh

**Affiliations:** Binghampton, N. Y.


### CLINICAL BUREAU.

### INTERMITTENT FEVER.

*Eupat. perf. Natrum mur.*

C. F. Millspaugh, M. D., Binghampton, N. Y.

Willie R., æt. 10, paludal environs; had called several times since 6 A. M. for cold water, of which he drank largely, At 7.30 a severe chill developed, during which he had kept his mother constantly bringing him drink; at 8.30 the chill passed off with vomiting spells, followed by heat, which lasted with thirst until noon. This attack was completed without sweat, and patient felt as well as ever during the rest of the day.

Note:—Although the case looked quite plain, still I had expected to find some bone pains. But there was no pain at all, any time in the paroxysm. I gave *Eupat. perf.*, thinking to fully develop the paroxysm.

2nd day:—Patient sat up all day, with no symptoms at all, except weakness. I had him taken up stairs, to the highest and best ventilated room in the house, and ordered all his drinking water to be thoroughly boiled and cooled for his use.

3rd day:—Same symptoms developed as before, with an additional symptom: I found him crying bitterly with severe pain in the back, legs and arms: he said his bones ached as if he had had them crushed. The chill had commenced in the back. This chill, as before, passed off with vomiting, and the fever came on with trembling in the back and limbs. Thirst continued, with no sweat after the fever.

Now my case was as pretty a one as could be desired: I gave him *Sac. Lac.*, every hour, until I called again.

4th day:—Called at 8 P. M., and prescribed *Eupat. perf.* 200 (D.), one dose, 10 pills, No. 8. and *Sac. Lac.*

5th day:—Chill slighter, nausea, but no vomiting. Paroxysm lasted only until 11 A. M. Prescription, *Sac. Lac.*

7th day:—No nausea nor vomiting, no pain, less thirst. Paroxysm over at 10 A. M. Continue *Sac. Lac.*

9th day:—Better in every way, except pain in the region of the spleen during paroxysm. Repeat *Eupat. perf.* 200 (D.) as before.

11th day:—Better still; gave *Sac. Lac.*

13th day:—No paroxysm. No return of the trouble.

Remarks:—This mode of developing characteristics, I judge, is an innovation: at least I never have heard of it before, and now I am in a quandary as to whether I did right in giving the $\theta$, or not. Still, the only thing I regret is, that I had no higher potency of the drug to exhibit.

GEORGE B., æt. 12, same surroundings, had had four paroxysms of chill, fever and sweat, without further symptoms, except thirst. In twenty hours I could see no indications for any of our remedies, and was quite nonplussed, until the parents let me out of my difficulty by saying that I must either give him quinine, or they would call in a doctor who would. I had not enough patients to afford to lose one, so taking some empty gelatine capsules from my pocket, I filled three of them with *Sacch. Lac.*, and ordered one every fifteen minutes, as the chill was coming on, and left.

2nd day:—A very hard chill came on at 11 A. M., with great thirst, which continued during all stages. During the fever he had a most violent headache, and complained that his eyes felt as if they were filled with "sharp salt." Temperature, 105.6; tongue was white-coated; taste bitter when taking food, salty when not; water, he complained, "tasted bad." No appetite.

Note:—Could you ask a better chance to try moonshine than this? I gave three capsules of *Sacch. Lac.*, into one of which I placed 10 pellets No. 8 of *Natrum Mur.* 200 (D.), and ordered one every hour, commencing as soon as the paroxysm was over.

3rd day:—Called at 3 P. M. found the patient sitting in the sun on the porch of his home. On inquiry I found that he had been somewhat feverish during the night, but nothing more. Gave him 5 capsules of *Sac. Lac.*, at one hour apart.

4th day:—Called at 11 A. M., but my bird had flown; he was at his studies, he had gone to school. No trouble since.

Comments:—I afterward told the parents that I had given their boy no quinine, and as my reason for deceiving them, I said, that under no circumstances would I treat a patient with any drug that I would not give my own family. If they thought I was right, then they might call me again in sickness; otherwise, I was satisfied. * * * * * I have carried their little daughter through an attack of typhoid fever since. O moonshine, thou art a jewel!

